# Altered activation in sensorimotor network after applying rTMS over the primary motor cortex at different frequencies

**DOI:** 10.1002/brb3.1670

**Published:** 2020-06-07

**Authors:** Xiaoyu Wang, Lingyu Li, Wei Wei, Tingting Zhu, Guo‐Feng Huang, Xue Li, Hui‐Bin Ma, Yating Lv

**Affiliations:** ^1^ Institute of Psychological Sciences Hangzhou Normal University Hangzhou China; ^2^ Zhejiang Key Laboratory for Research in Assessment of Cognitive Impairments Hangzhou China; ^3^ Shandong Huayu University of Technology Dezhou China; ^4^ School of Information and Electronics Technology Jiamusi University Jiamusi China; ^5^ Integrated Medical Research School Jiamusi University Jiamusi China

**Keywords:** finger‐tapping task, functional MRI, primary motor cortex, repetitive transcranial magnetic stimulation, sensorimotor network

## Abstract

**Introduction:**

Repetitive transcranial magnetic stimulation (rTMS) over the primary motor cortex (M1) can modulate brain activity both in the stimulated site and remote brain areas of the sensorimotor network. However, the modulatory effects of rTMS at different frequencies remain unclear. Here, we employed finger‐tapping task‐based fMRI to investigate alterations in activation of the sensorimotor network after the application of rTMS over the left M1 at different frequencies.

**Materials and Methods:**

Forty‐five right‐handed healthy participants were randomly divided into three groups by rTMS frequency (HF, high‐frequency, 3 Hz; LF, low‐frequency, 1 Hz; and SHAM) and underwent two task‐fMRI sessions (RH, finger‐tapping with right index finger; LH, finger‐tapping with left index finger) before and after applying rTMS over the left M1. We defined regions of interest (ROIs) in the sensorimotor network based on group‐level activation maps (pre‐rTMS) from RH and LH tasks and calculated the percentage signal change (PSC) for each ROI. We then assessed the differences of PSC within HF or LF groups and between groups.

**Results:**

Application of rTMS at different frequencies resulted in a change in activation of several areas of the sensorimotor network. We observed the increased PSC in M1 after high‐frequency stimulation, while we detected the reduced PSC in the primary sensory cortex (S1), ventral premotor cortex (PMv), supplementary motor cortex (SMA), and putamen after low‐frequency stimulation. Moreover, the PSC in the SMA, dorsal premotor cortex (PMd), and putamen in the HF group was higher than in the LF group after stimulation.

**Conclusion:**

Our findings suggested that activation alterations within sensorimotor network are dependent on the frequency of rTMS. Therefore, our findings contribute to understanding the effects of rTMS on brain activation in healthy individuals and ultimately may further help to suggest mechanisms of how rTMS could be employed as a therapeutic tool.

## Graphical abstract

Repetitive transcranial magnetic stimulation (rTMS) over the primary motor cortex (M1) could modulate the activity both in the stimulated site and remote brain regions of the sensorimotor network. We employed finger‐tapping task‐based fMRI to investigate the altered activations of the sensorimotor network after the application of rTMS over the left M1 at different frequencies.

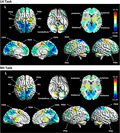

## 1 INTRODUCTION

Repetitive transcranial magnetic stimulation (rTMS) is a painless, noninvasive brain stimulation technique, which could modulate cortical activity and has been increasingly employed both in clinical practice and in research for the treatment of patients with motor dysfunctions, such as stroke or Parkinson's disease (Chung et al., 2019; Hallett, 2007; Kim et al., 2006; Lefaucheur et al., 2014; Takeuchi, Chuma, Matsuo, Watanabe, & Ikoma, 2005). Application of rTMS over the primary motor cortex (M1) at different frequencies induced either excitatory or inhibitory effects on the cortical activity: High‐frequency rTMS (> 1 Hz) increased cortical activity in the ipsilateral hemisphere (Pascual‐Leone, Amedi, Fregni, & Merabet, 2005; Peinemann et al., 2004; Wassermann, 1998), whereas low‐frequency rTMS (≤ 1 Hz) has been shown to decrease activity in the ipsilateral side and increase activity in the contralateral hemisphere (Muellbacher, Ziemann, Boroojerdi, & Hallett, 2000; Wassermann, 1998; Ziemann, 2004). Moreover, previous studies have suggested that rTMS over M1 is capable of modulating activity not only in the stimulated site (Baudewig et al., 2001; Bestmann, Baudewig, Siebner, Rothwell, & Frahm, 2003; Rounis et al., 2005) but also in remote areas of the sensorimotor network (Bestmann et al., 2003; Bestmann, Baudewig, Siebner, Rothwell, & Frahm, 2004; Yoo et al., 2008).

Noninvasive neuroimaging techniques serve as promising tools to identify functional and structural alterations induced by rTMS in the entire brain (Bohning et al., 1999; Fox et al., 2006; Min et al., 2016; Rounis et al., 2005). Functional magnetic resonance imaging (fMRI), one of the neuroimaging techniques, measures the blood oxygenation level‐dependent (BOLD) signal, which is highly correlated with neuronal activity of the brain, and has been widely employed to investigate the intrinsic brain functions at resting state (Biswal et al., 2010; Biswal, Zerrin Yetkin, Haughton, & Hyde, 1995) or to localize brain involvement in cognitive tasks (Mehler et al., 2019; Phan, Wager, Taylor, & Liberzon, 2002; Stoodley, Valera, & Schmahmann, 2012). Using fMRI technique, Yoo and colleagues found significantly increased activation in presupplementary motor cortex (SMA) and ipsilateral cerebellum after high‐frequency rTMS (10 Hz) over the right M1 (Yoo et al., 2008). Min and colleagues observed that the participants who received low‐frequency rTMS (1 Hz) over the left M1 showed decreased activation in the ipsilateral primary sensory cortex (S1) and SMA, and increased degree of the deactivation in contralateral S1 when performing a finger‐tapping task (Min et al., 2016). However, these studies predominantly focused on the alterations of brain activation induced by application of rTMS at a specific frequency, and the similarities and differences of the changed activation between high‐frequency and low‐frequency rTMS were rarely investigated. To the best of our knowledge, there has only one study which employed positron emission tomography (PET) to assess the regional cerebral blood flow (rCBF) changes during a paced free selection of finger movements task after application of rTMS over the left M1 at different frequencies (Rounis et al., 2005). The author observed an increased rCBF in the left M1 after stimulation at both high (5 Hz) and low (1 Hz) frequencies, whereas they found a different effect on the ipsilateral central sulcus; low‐frequency stimulation resulted in an increased rCBF, while high‐frequency stimulation decreased the rCBF. However, it was not clear whether these alterations induced by rTMS at different frequencies can be observed using the BOLD signal as well. Therefore, a more in‐depth comparison of the effects of high‐frequency and low‐frequency rTMS over M1 was required.

Moreover, previous studies have focused merely on the alterations of the brain activation during the finger movement contralateral to the stimulated M1. Nevertheless, accumulating evidence from clinical researches reveals that the sensorimotor areas affected by the brain disorders can show altered brain activation during ipsilateral hand movement. For example, Cramer and colleagues found the stroke patients whose lesion areas involved the sensorimotor network exhibited a decreased activation in the unaffected hemisphere, including precentral and postcentral gyrus, when they performed a motor task with the hand ipsilateral to the lesion side (Cramer et al., 1997). Similar findings were also observed in multiple sclerosis patients (Lee et al., 2000). However, the potential changes in activation of sensorimotor areas during the finger movement ipsilateral to the stimulated M1 are still unclear.

In this study, we employed finger‐tapping task‐based fMRI to investigate the alterations in activation of sensorimotor areas after the application of rTMS over the left M1. Specifically, we sought to determine whether and how different frequencies of rTMS affect the BOLD signals of the sensorimotor areas during the motor task with hands both contralateral and ipsilateral to the stimulated M1. We hypothesized that the high‐frequency rTMS increases activation of the sensorimotor network, while low‐frequency rTMS would decrease activation.

## 2 MATERIALS AND METHODS

### 2.1 Participants

Forty‐five healthy participants (mean age: 23 ± 2.67 years, 25 females and 20 males) were recruited from local universities in this study. All the participants were right‐handed and had no history of neurological or psychiatric disorders. The participants were randomly divided into three groups based on rTMS frequency: the high‐frequency (HF) group (*n* = 15, mean age: 24 ± 2.56 years, 8 females and 7 males), low‐frequency (LF) group (*n* = 15, mean age: 22.8 ± 3.10 years, 8 females and 7 males), and the sham group (SHAM, *n* = 15, mean age: 22.4 ±  2.16 years, 9 females and 6 males). This study was approved by the Ethics Committee of the Center for Cognition and Brain Disorders in Hangzhou Normal University. Written informed consent was obtained from each participant.

### 2.2 Data acquisition

Each participant underwent one MR scan before (pre‐rTMS) and one scan after rTMS stimulation (post‐rTMS) using the same scanning protocol. The second MR scan was performed within 30 min after stimulation (mean time: 14 ± 4.83 min, HF, 13.93 ± 4.30 min; LF, 14.67 ± 5.01 min; SHAM, 12.40 ± 5.18 min) to ensure measurement of stimulation effects (Siebner et al., 2009). The MRI data were acquired using a GE 3‐Tesla scanner (MR‐750, GE Medical Systems, Milwaukee, WI) located at the Affiliated Hospital of Hangzhou Normal University. Each MR scan included two fMRI sessions with the finger‐tapping task and one high‐resolution T1‐weighted structural MRI data.

The fMRI data were acquired using an echo‐planar imaging sequence: 43 axial slices, repetition time (TR) = 2,000 ms, echo time (TE) = 30 ms, field of view (FOV) = 220 × 220 mm^2^, voxel size = 3.44 mm × 3.44 mm × 3.20 mm, and flip angle = 60°. Each participant underwent two fMRI sessions: a left index finger‐tapping task session (LH task) and a right index finger‐tapping task session (RH task). The order of the two sessions was counterbalanced across participants. For each session, participants completed a block‐designed finger‐tapping task with eight 20 s task blocks and seven 20 s rest blocks. During the finger‐tapping block, participants were presented with a visual stimulus (red circle) flashing at a frequency of 1 Hz and instructed to press a key with their index finger following this stimulus, while they were instructed to keep their gaze at a white cross presented in the middle of the screen during the resting block. Each session consisted of 150 contiguous volumes and lasted for 5 min.

The structural MRI data were acquired using a 3D‐MPRAGE sequence: 176 sagittal slices, TR = 8,100 ms, TE = 3.1 ms, FOV = 256 × 256 mm^2^, and voxel size = 1 mm × 1 mm × 1mm.

### 2.3 rTMS intervention

For each participant, rTMS was delivered over the left M1 using a Magstim TMS machine (Magstim Inc.) equipped with a figure‐of‐eight coil. The application of rTMS was carried out following the safety guidance provided by the International Workshop on the Safety of Repetitive Transcranial Magnetic Stimulator (Wassermann, 1998).

#### 2.3.1 Resting motor threshold (RMT)

Participants were instructed to sit comfortably in an adjustable armchair. Motor evoked potential (MEP) amplitudes were recorded from abductor pollicis brevis (APB) muscle of their right hand. We then located the left M1 based on the “hand knob” area of the structural MR image of each participant and marked coordinates individually using the Brainsight software (https://www.rogue‐research.com/tms/brainsight‐tms). Frameless stereotaxy was then applied to coregister the structural image to the head for participants (Paus et al., 1997). Each participant's head position was assessed using the Polaris infrared tracking system (Northern Digital, Waterloo, Canada) based on four landmarks (nasion, nose tip, and intertragal notch of both ears). Single‐pulse TMS was delivered to target position while subsequently moving the coil systematically in 1‐cm increments at a constant suprathreshold stimulus intensity to detect the “hot spot” (i.e., the location where MEP could be evoked with highest amplitude and shortest latencies) (Cárdenas‐Morales et al., 2014; Yoo et al., 2008). The RMT was defined as the lowest stimulus intensity that elicited at least five responses ≥ 50 µV within 10 consecutive single pulses over the “hot spot” (Liang et al., 2018; Rossini et al., 1994; Rothwell et al., 1999).

#### 2.3.2 Location of the individual rTMS target

Prior to applying rTMS, we projected the activation map of the RH task (pre‐rTMS, see the paragraph of generation of activation maps for more details) to the anatomical image using the Brainsight software. The most activated voxel in the left M1 (anterior wall of central sulcus) was then localized as the individual rTMS target for each participant.

#### 2.3.3 rTMS protocol

The coil was placed tangentially over the target region. rTMS was then delivered with a pulse magnitude set at 90% of the RMT (Min et al., 2016; Rounis et al., 2005). Each participant received a total of 1,500 pulses.

HF group: The participants in HF group received 5 successive pulse blocks interspersed with 15 s quitting time. Each block was composed of 300 pulses at a frequency of 3 Hz and lasted for 100 s. The session lasted 9.3 min.

LF group: Low‐frequency rTMS was also administered in 5 consecutive pulse blocks interspersed with 15 s quitting time. Each block consisted of 300 pulses at a frequency of 1 Hz and lasted for 300 s. The session lasted 26 min.

SHAM group: To reduce the possible cortical stimulation effects, the coil was placed at a degree of 90° to the skull for sham group (Herwig, Cardenas‐Morales, Connemann, Kammer, & Schönfeldt‐Lecuona, 2010; Nettekoven et al., 2014). Otherwise, the stimulation parameters were identical to the LF group. 

### 2.4 Data analysis

#### 2.4.1 Data preprocessing

The fMRI data were preprocessed in the native space using Data Processing & Analysis for Brain Imaging (DPABI) (Yan, Wang, Zuo, & Zang, 2016) including (a) slice timing to correct for differences in image acquisition time between slices; (b) head motion correction; and (c) spatial smoothing with an isotropic Gaussian kernel with a full width at half maximum (FWHM) of 6 mm. No participants were excluded from further analysis due to large head motion (more than 2.0 mm of maximal translation in any direction of *x*, *y*, or *z* or 2.0° of maximal rotation throughout the course of scanning).

#### 2.4.2 Generation of activation maps

The fMRI data for each session in pre‐rTMS were further processed to generate the individual‐level activation maps by using a general linear model in SPM12 (http://www.fil.ion.ucl.ac.uk/spm/). The onset time and duration of the finger‐tapping blocks were convolved with the hemodynamic response function and modeled as regressors in design matrix. The six head motion parameters were additionally included as variables of no interest to eliminate the influence of head motion in the design matrix, and the contrast image was then calculated. For each participant, we obtained two contrast images (LH and RH tasks). The contrast image for RH task was also used to localized the individual rTMS target.

Each contrast image was further spatially normalized to the Montreal Neurological Institute (MNI) space via the deformation fields derived from the tissue segmentation of the structural image, which has been coregistered to the mean functional images for each task‐fMRI session. Voxel‐wise one‐sample *t* tests against the null hypothesis of zero magnitude were performed to obtain the group‐level activation maps of all participants (*n* = 45, pre‐rTMS) for both the left (LH task) and right (RH task) index finger‐tapping tasks. We corrected for multiple comparisons using a false discovery rate (FDR) correction (*p* < .05).

#### 2.4.3 Definition of regions of interest (ROIs)

We first selected 18 sensorimotor regions which were symmetrically distributed in both hemispheres including six cortical regions and three subcortical regions (Bestmann et al., 2004; Denslow, Lomarev, George, & Bohning, 2005; Hanakawa et al., 2009; L. Lee et al., 2003). The regions were defined according to the Brodmann area (BA) and Anatomical Automatic Labeling (AAL) (Tzourio‐Mazoyer et al., 2002) atlases (Table 1). The premotor cortex was divided into ventral premotor (PMv) and dorsal premotor (PMd) by excluding all voxels between sagittal “*x*” coordinates −13 and 13 and splitting along the horizontal “*z*” coordinate 48 into the PMv (*z* < 48) and PMd (*z* ≥ 48) (Tomassini et al., 2007; Valchev et al., 2015). For each task session, the peak voxel (most activated or deactivated in group‐level activation map) was selected as the seed voxel in each region. Eighteen spherical ROIs (radius = 5 mm), centered at seed voxels, were then generated for LH or RH tasks separately (Table 1).

**TABLE 1:** Motor‐related regions from pre‐rTMS group‐level activation maps (*n* = 45)

Brain regions	AAL/BA	LH				RH			
		Peak MNI coordinates				Peak MNI coordinates			
		*x*	*y*	*z*	*t*_max_	*x*	*y*	*z*	*t*_max_
L_Primary motor	4	−27	−33	66	−10.01	−42	−15	57	10.81
R_Primary motor	4	36	−18	51	12.55	12	−42	69	−10.79
L_Primary sensory	1/2/3	−27	−45	66	−13.15	−39	−21	51	9.66
R_Primary sensory	1/2/3	36	−21	51	12.55	27	−42	63	−12.06
L_dorsal premotor cortex	6/44	−51	0	51	8.88	−39	−15	54	10.8
R_dorsal premotor cortex	6/44	39	−15	54	11.52	27	−27	63	−8.44
L_ventral premotor cortex	6/44	−12	6	48	5.65	−51	0	48	7.68
R_ventral premotor cortex	6/44	15	9	48	4.78	15	9	48	2.55
L_Supplementary motor cortex	Supp_Motor _Area_L	−6	0	60	11.72	−3	0	57	10.74
R_Supplementary motor cortex	Supp_Motor _Area_R	6	3	63	9.43	3	3	63	7.8
L_Cingulate motor cortex	24/32	−6	6	48	6.92	−3	9	51	6.55
R_Cingulate motor cortex	24/32	9	9	48	6.79	3	9	51	5.73
L_Putamen	Putamen_L	−24	−6	9	5.83	−27	−3	3	6.76
R_Putamen	Putamen_R	27	−3	9	6.1	24	9	0	5.12
L_Thalamus	Thalamus_L	−21	−27	0	−10.65	−15	−18	6	7.4
R_Thalamus	Thalamus_R	15	−18	3	7.88	21	−24	0	−6.62
L_Cerebellum	Cerebelum_4_5_L	−15	−51	−21	14.14	—	—	—	—
	Cerebelum_6_L	—	—	—	—	−27	−57	−27	6.86
R_Cerebellum	Cerebelum_4_5_R	—	—	—	—	15	−51	−21	13.21
	Cerebelum_6_R	27	−57	−27	7.52	—	—	—	—

Abbreviations: AAL, Anatomical Automatic Labeling atlases; BA, Brodmann area; L, left hemisphere; LH, left index finger‐tapping task; R, right hemisphere; RH, right index finger‐tapping task.

#### 2.4.4 Percentage signal change (PSC) of BOLD signal

The preprocessed functional data were spatially normalized to the MNI space via the deformation fields derived from tissue segmentation of the T1 images, which had been coregistered to the mean functional images for each task‐fMRI session. For each participant, we extracted the averaged BOLD signal of each ROI for each task session and then calculated the PSC of each averaged BOLD signal. PSC of the BOLD signal was defined as follows:

$$\text{PSC}=\frac{\text{Activation}\;\text{signal}-\text{Baseline}\;\text{signal}}{\text{Baseline}\;\text{signal}}$$

$$\text{Activation}\;\text{signal}=\frac{\sum_{i=1}^{N}\text{time}\;{\text{point}}_{i}\;\text{of}\;\;\text{task}\;\text{blocks}}{N}$$

$$\text{Baseline}\;\text{signal}=\frac{\sum_{i=1}^{N}\text{time}\;{\text{point}}_{i}\;\text{of}\;\text{resting}\;\text{blocks}}{N}$$

For each block, the first three time points (6 s) were discarded to remove the effect of the transition phase during the rise and fall of the BOLD signal. Thus, 7 time points (14 s) were left in each block for PSC calculation. We also discarded the last task block, where *N* was the total number of time points left in task or resting blocks (here, *N* = 49); *i* represented the *i*th time point.

### 2.5 Statistical analysis

We tested between‐group differences in age, sex, and the time intervals between rTMS and subsequent MR scan with the Statistical Package for the Social Sciences (SPSS; SPSS Inc.). Differences in age and time intervals among three groups were assessed using one‐way analysis of variance (one‐way ANOVA). Sex differences were quantified using the Pearson chi‐square test.

Using SPSS software, we performed two‐way repeated measures analysis of variance (ANOVA) for PSC of each ROI with three levels (HF, LF, and SHAM groups) as the between‐subject factor and two levels (pre‐ and post‐rTMS conditions) as the within‐subject factor. *Post hoc* comparisons were then performed in those PSC with significant interactions (frequency of rTMS × MR session) to explore the effect of rTMS at different frequencies.

To fully address the alterations in activation of sensorimotor areas after the application of rTMS, between‐group differences in PSC of BOLD signals for each ROI after the application of rTMS were further inferred using two‐sample *t* tests (HF group vs. SHAM group, LF group vs. SHAM group, and HF group vs. LF group); within‐group (HF group and LF group) differences in PSC for each ROI were inferred with paired *t* tests (pre‐rTMS condition vs. post‐rTMS condition). A threshold of *p* < .05 (two‐tailed) was considered statistically significant.

## 3 RESULTS

### 3.1 Demographic characteristics

No significant differences were found in age (*F*_2,12_ = 0.855, *p* = .433), sex (*Χ* ^2^ = 0.180, *p* = .914), or time intervals (between rTMS and subsequent MRI scan) (*F*_2,12_ = 1.496, *p* = .236) among the three groups.

### 3.2 Task activations

**FIGURE 1:**
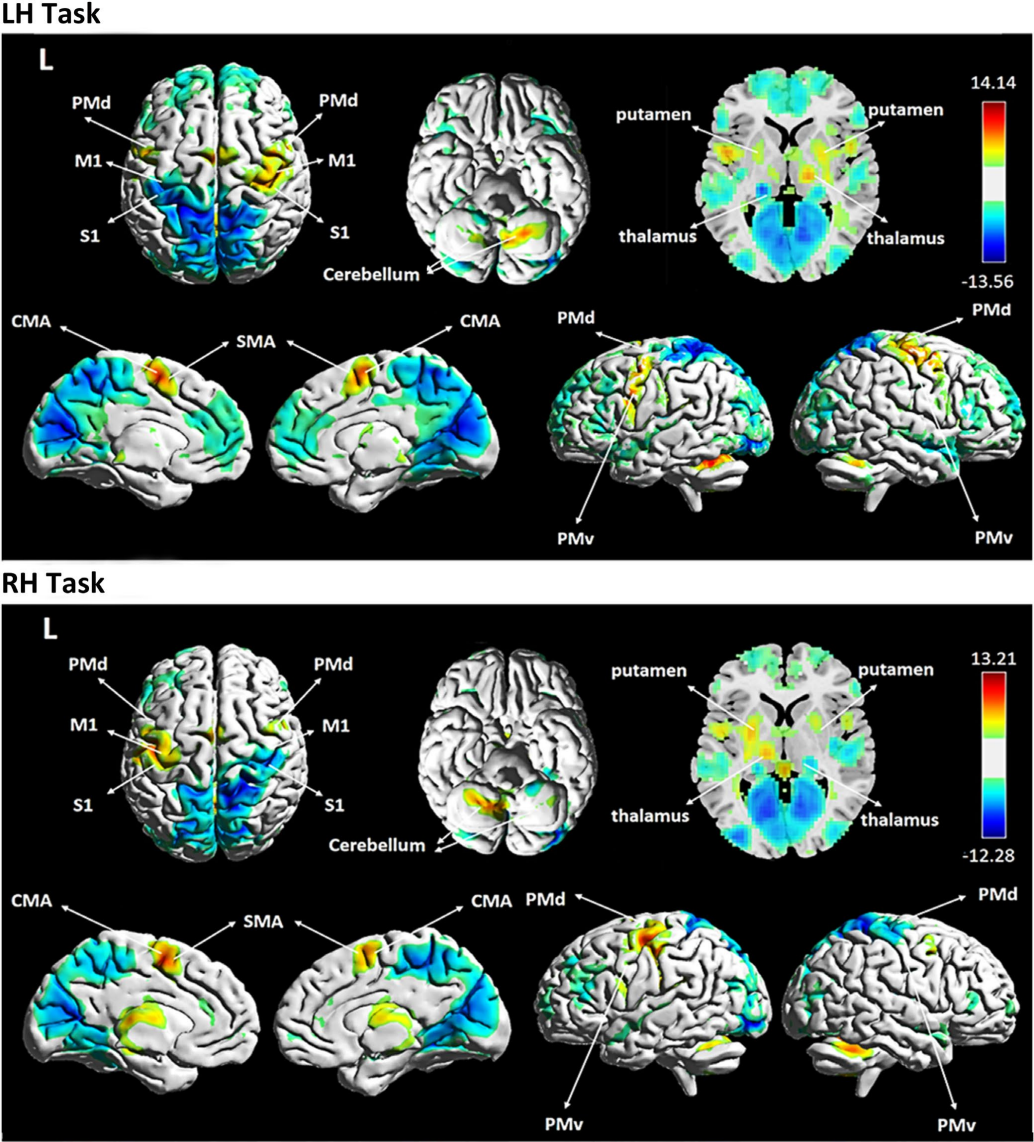
One‐sample *t* tests generated from 45 participants (pre‐rTMS condition, *p* < .05, FDR correction for multiple comparisons). LH task, activation maps of the left index finger‐tapping task; RH task, activation maps of the right index finger‐tapping task

As expected, one‐sample *t* tests (*p* < .05, FDR correction) of all participants showed that both LH and RH tasks showed similar activation patterns: significant activation was found in several cortical regions, including the contralateral M1, S1, PMd, and bilateral SMA, and subcortical regions, such as the contralateral thalamus and putamen, while significant deactivations were observed in the ipsilateral M1 and S1 (Figure 1).

### 3.3 Differences in PSC

#### 3.3.1 Two‐way repeated measures ANOVA

During the RH task, we found the PSC of the right PMd exhibited the significant main effect of MR session (*F*_1,42_ = 6.480, *p* = .015) and the PSC of the right SMA exhibited the significant main effect of frequency (*F*_1,42_ = 4.504, *p* = .017). During the LH task, the PSC of the left PMd (*F*_1,42_ = 4.981, *p* = .031) and PMv (*F*_1,42_ = 6.117, *p* = .018) showed significant main effects of MR session, while the right PMv exhibited the significant main effect of frequency (*F*_1,42_ = 3.327, *p* = .046).

However, no significant interaction (frequency of rTMS × MR session) was observed in PSC of each ROI.

#### 3.3.2 Differences within the HF group

**FIGURE 2:**
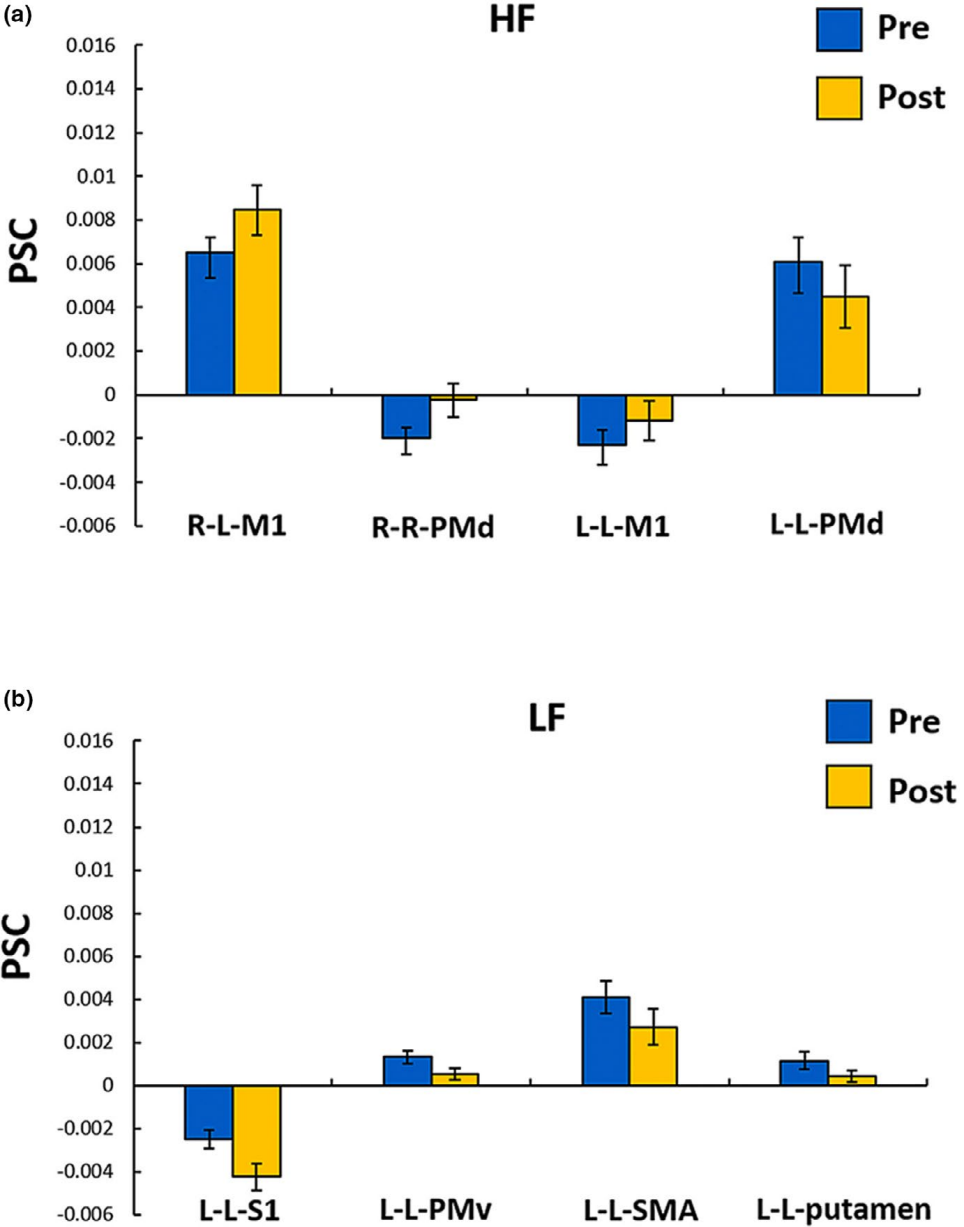
The regions which showed significant PSC differences between pre‐rTMS and post‐rTMS in the HF group (a) and the LF group (b). R‐L areas, the first and second letters represent the right index finger and left brain hemisphere, respectively; HF, high‐frequency; LF, low‐frequency; pre, pre‐rTMS condition; post, post‐rTMS condition; PMv, ventral premotor cortex; PMd, dorsal premotor cortex; S1, primary sensory cortex; M1, primary motor cortex; and SMA, supplementary motor cortex. Error bars represent one strand error of the mean

For the RH task, the PSC of the left M1 (mean ± *SD*: pre‐rTMS = 0.0065 ± 0.0027, post‐rTMS = 0.0085 ± 0.0044, *t* (14) = −2.195, *p* = .046, Cohen's *d*_z_ = 0.570) and right PMd (deactivation) (mean ± *SD*: pre‐rTMS = −0.0020 ± 0.0018, post‐rTMS = −0.0002 ± 0.0030, *t* (14) = −2.435, *p* = .029, Cohen's *d*_z_ = 0.630) was significantly increased after rTMS. For the LH task, the PSC of the left M1 (deactivation) (mean ± *SD*: pre‐rTMS = −0.0023 ± 0.0028, post‐rTMS = −0.0012 ± 0.0035, *t* (14) = −2.386, *p* = .032, Cohen's *d*_z_ = 0.620) was significantly increased after the stimulation, while a significantly decreased PSC was found in the left PMd (mean ± *SD*: pre‐rTMS = 0.0061 ± 0.0044, post‐rTMS = 0.0045 ± 0.0056, *t* (14) = 2.338, *p* = .035, Cohen's *d*_z_ = 0.580) when compared with pre‐rTMS condition (Figure 2a).

#### 3.3.3 Differences within the LF group

Compared with the pre‐rTMS condition, the PSC of the left S1 (deactivation) (mean ± *SD*: pre‐rTMS = −0.0025 ± 0.0017, post‐rTMS = −0.0043 ± 0.0023, *t* (14) = 2.294, *p* = .038, Cohen's *d*_z_ = 0.590), PMv (mean ± *SD*: pre‐rTMS = 0.0013 ± 0.0011, post‐rTMS = 0.0005 ± 0.0011, *t* (14) = 2.334, *p* = .035, Cohen's *d*_z_ = 0.600), SMA (mean ± *SD*: pre‐rTMS = 0.0041 ± 0.0029, post‐rTMS = 0.0027 ± 0.0033, *t* (14) = 2.679, *p* = .018, Cohen's *d*_z_ = 0.690), and putamen (mean ± *SD*: pre‐rTMS = 0.0011 ± 0.0016, post‐rTMS = 0.0004 ± 0.0011, *t* (14) = 2.168, *p* = .048, Cohen's *d*_z_ = 0.560) was significantly decreased in LH task after the stimulation (Figure 2b). No significant changes were found in the RH task.

#### 3.3.4 Differences between the HF group and SHAM group

**FIGURE 3:**
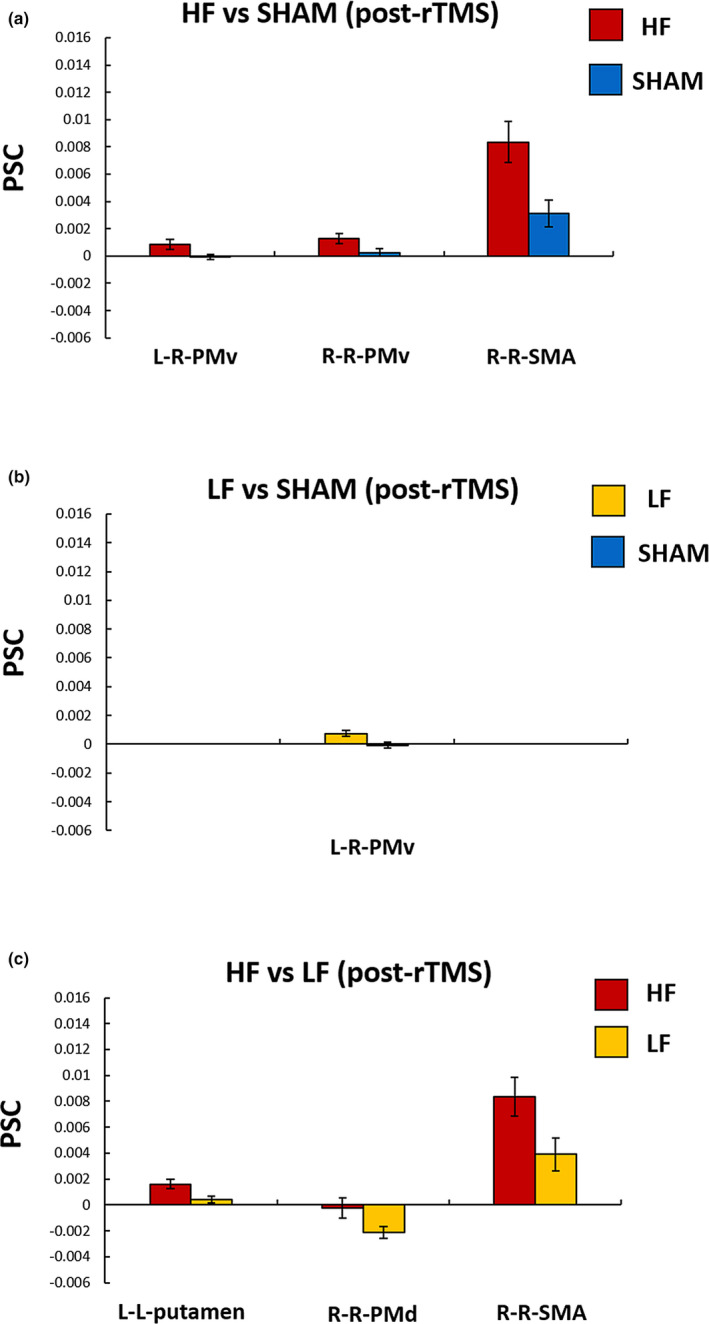
The regions which showed significant differences in PSC after the rTMS application between groups: (a) HF versus SHAM group, (b) LF versus SHAM group, and (c) HF versus LF group. L‐R areas, the first and second letters represent the left index finger and right brain hemisphere, respectively; HF, high‐frequency group; LF, low‐frequency group; PMv, ventral premotor cortex; PMd, dorsal premotor cortex; and SMA, supplementary motor cortex. Error bars represent one strand error of the mean

The PSC of the right PMv (mean ± *SD*: HF = 0.0013 ± 0.0015, SHAM = 0.0003 ± 0.0011, *t* (28) = 2.158, *p* = .040, Cohen's *d*_s_ = 0.790) and SMA (mean ± *SD*: HF = 0.0084 ± 0.0058, SHAM = 0.0031 ± 0.0038, *t* (23.894) = 2.929, *p* = .007, Cohen's *d*_s_ = 1.070) was significantly increased during the RH task of the HF group compared with the SHAM group. For the LH task, the PSC of the right PMv (mean ± *SD*: HF = 0.0009 ± 0.0014, SHAM = 0.0001 ± 0.0008, *t* (22.543) = 2.278, *p* = .031, Cohen's *d*_s_ = 0.830) was also increased significantly in the HF group compared with the SHAM group (Figure 3a).

#### 3.3.5 Differences between the LF group and SHAM group

The PSC of the right PMv (mean ± *SD*: LF = 0.0007 ± 0.0008, SHAM = 0.0001 ± 0.0008, *t* (28) = 2.756, *p* = .010, Cohen's *d*_s_ = 1.010) was significantly increased during the LH task of the LF group compared with the SHAM group (Figure 3b), which was consistent with the findings in the HF group.

#### 3.3.6 Differences between the HF group and LF group

The HF group exhibited a higher PSC in the right PMd (deactivation) (mean ± *SD*: HF = −0.0002 ± 0.0030, LF = −0.0021 ± 0.0018, *t* (22.949) = 2.098, *p* = .047, Cohen's *d*_s_ = 0.770) and SMA (mean ± *SD*: HF = 0.0084 ± 0.0058, LF = −0.0039 ± 0.0048, *t* (28) = 2.277, *p* = .031, Cohen's *d*_s_ = 0.830) during the RH task and the left putamen (mean ± *SD*: HF = 0.0016 ± 0.0014, LF = 0.0004 ± 0.0011, *t* (28) = 2.529, *p* = .018, Cohen's *d*_s_ = 0.920) during the LH task (Figure 3c).

### 3.4 Voxel‐based group differences analyses

To validate our findings of the ROI‐based analyses, we also performed voxel‐based analyses on the alterations of activation during LH task and RH task within sensorimotor network. We found that within‐group differences (Figures S1 and S2) and between‐group differences (Figures S3–S5) remained largely unchanged. The methods and results of these voxel‐based analyses are provided in the Supplementary Materials.

## 4 DISCUSSION

In this study, we employed finger‐tapping task‐based fMRI to investigate the alterations in activation of the sensorimotor areas induced by rTMS over M1 at different frequencies. We found that changes in activation were associated with the frequency of stimulation. This occurred predominantly in five areas: the M1, S1, SMA, premotor cortex, and putamen. Overall, these findings may contribute to elucidating alterations in brain function after application of rTMS at different frequencies and help us to understand the potential mechanisms of how rTMS could be used for treatment of patients with motor dysfunctions.

The activation of the left M1, the stimulated site, was significantly increased after high‐frequency stimulation during both LH and RH tasks. Our results provide further evidence for an increased cortical activity in the ipsilateral hemisphere after application of high‐frequency rTMS (Bestmann et al., 2003, 2004). In contrast, no significant changes in the left M1 were found after low‐frequency stimulation. As there may be dose‐dependent effects of rTMS stimulation, we hypothesize that the lack of a significant effect of low‐frequency rTMS in stimulated M1 may be the short series of stimuli. Therefore, it would be interesting to explore whether prolonged stimulation time can induce alterations in activation of the M1 region in future studies.

Comparing to activation before stimulation, the degree of deactivation of the left S1 was increased in LF group during the LH task. S1, located in the postcentral gyrus, plays a fundamental role in somatosensation and control of action (Sarfeld et al., 2012; Valchev et al., 2015). S1 has direct anatomical connections with the M1, and thus likely to be affected indirectly by M1 rTMS stimulation (Denslow et al., 2005; Hanakawa et al., 2009; Min et al., 2016). Previous studies suggested that intracortical M1‐sensory connections may account for the spread of inhibitory modulation from M1 to S1 after low‐frequency rTMS stimulation (Min et al., 2016). However, the HF group did not show any significant alterations in S1 after stimulation in either task. These results were therefore not consistent with previous studies which observed significant reduction in activation of the S1 area in a sensory perception task after application of high‐frequency rTMS (Yoo et al., 2008). While the differences between the two tasks (sensory perception rather than finger‐tapping) might have affected the results, it would be interesting to explore activation alterations in S1 during different sensorimotor tasks after application of rTMS in future studies.

The activation of the left SMA was decreased during the LH task after low‐frequency rTMS stimulation, while the activation of the right SMA was increased during the RH task after high‐frequency rTMS stimulation. As shown in Figure 1, the SMA‐ROI, located in the posterior part of the SMA, is commonly referred to as SMA‐proper (Bestmann et al., 2003; Picard & Strick, 2001) which is functionally and anatomically interconnected with M1 (Geyer, Matelli, Luppino, & Zilles, 2000; Grefkes, Eickhoff, Nowak, Dafotakis, & Fink, 2008; Sarfeld et al., 2012). The SMA‐proper is mainly involved in processing of relatively simple hand movements (Luppino, Matelli, Camarda, & Rizzolatti, 1993; Tanji & Shima, 1994). After application of low‐frequency rTMS, we found a decreased activation of the left M1 during the LH task. Although this decrease did not reach significance (mean ± *SD*: pre‐rTMS = −0.0019 ± 0.0019, post‐rTMS = −0.0032 ± 0.0026), it might result in decreased activation of the ipsilateral SMA‐proper due to the intrahemispheric coupling between these two regions (Cárdenas‐Morales et al., 2014; Sarfeld et al., 2012). However, we observed an increased activation of the left M1 during RH task (mean ± *SD*: pre‐rTMS = 0.0065 ± 0.0027, post‐rTMS = 0.0085 ± 0.0044) after high‐frequency stimulation. Thus, we propose that the increased activation of the right SMA‐proper during the RH task after application of high‐frequency rTMS might be attributed to positive coupling between the right SMA and left M1 during movements of the right hand (Cárdenas‐Morales et al., 2014). As the effects of rTMS might be influenced by interhemispheric interactions between the SMA and M1, future studies are required to elucidate the connections between these two regions.

Like SMA, similar effects of rTMS at different frequencies were observed in the ventral premotor cortex (PMv): Activation of the left PMv was decreased during LH task after low‐frequency rTMS, while activation of right PMv was increased during RH task after high‐frequency rTMS. The PMv is involved in processing information for grasping objects and shaping the hand posture appropriately prior to attempting a grasp (Davare, 2006; Fiori et al., 2017; Quessy, Côté, Hamadjida, Deffeyes, & Dancause, 2016). It is worth noting that significant intrahemispheric coupling between SMA and PMv has been consistently observed (Cárdenas‐Morales et al., 2014; Moulton et al., 2017). Thus, we conjectured that these alterations of activation in PMv may be due to the changes in SMA after rTMS. In addition, we found an increased activation of right PMv during the LH task after receiving stimulation at both high and low frequencies, which is consistent with findings of a previous study. Rounis and colleagues observed increased rCBF in the right PMv after application of rTMS over left M1 at both high and low frequencies, by employing the conjunction analysis (Rounis et al., 2005). Our results implied a physiological basis of these activity alterations: Increased activation of the contralateral PMv during a finger movement ipsilateral to the stimulated M1 was related to high rates of metabolism and CBF. Future studies utilizing both CBF and BOLD signal may elucidate their relationships.

In another premotor area, the dorsal premotor cortex (PMd), high‐frequency rTMS induced distinct effects in different tasks: We found an increased activation of the right PMd during the RH task, but decreased activation of the left PMd during the LH task. The PMd contains a high proportion of cells that respond to sensory and motor cues (Weinrich & wise, 1982) and plays an important role in the sensorimotor integration and movement selection (Moisa, Siebner, Pohmann, & Thielscher, 2012). L. Côté and colleagues showed that the ipsilateral PMd could induce a powerful inhibitory effect on M1, while the contralateral PMd exerted a facilitatory effect on M1 (L. Côté, Hamadjida, Quessy, & Dancause, 2017). We therefore speculated that the decreased activation of the left PMd during LH task and the increased activation of the right PMd during the RH task after high‐frequency rTMS may reduce the inhibitory effect and increase the facilitatory effect on left M1 simultaneously, which results in increased activation on the stimulated site. Accordingly, we observed an increased activation of the left M1 during both LH and RH tasks after high‐frequency stimulation. These findings further demonstrated the regulatory role of bilateral PMd in activation alterations of the M1.

The activation of ipsilateral putamen was decreased during the LH task after the low‐frequency rTMS stimulation. The putamen, one part of basal ganglia, is responsible for the execution of a relatively simple and unprepared hand movements (Gerardin et al., 2004). Previous studies indicated strong functional connections between the putamen and M1 (Simioni, Dagher, & Fellows, 2016; Wu, Hallett, & Chan, 2014). Thus, we proposed that the decreased activation of the putamen may at least partly contribute to the decreased activation of the left M1 after low‐frequency rTMS stimulation. Significant changed activation of putamen also provided further evidence that rTMS could induce changed activation of subcortical areas by stimulation of cortical regions (Bestmann et al., 2004; Wang et al., 2014; Yoo et al., 2008), which may provide insights into the prospective rTMS treatment of pathological states like Parkinson's disease.

Employing resting‐state fMRI data to investigate topological alterations in the sensorimotor network, the participants included in current study were also demonstrated reduced nodal betweenness in the right SMA after high‐frequency stimulation and reduced nodal degree and betweenness centrality in the left paracentral lobule (PCL) after application of low‐frequency rTMS (Wei et al., 2019). The activation alterations in the SMA were also observed in the current study, which may indicate that the SMA is a key structure for high‐frequency rTMS, affecting the information flow in the sensorimotor network. Although we did not find activation alterations in the left PCL, the left M1, which showed a decreased functional connectivity with the left PCL in our previous study (Wei et al., 2019), also exhibited reduced activation after application of low‐frequency rTMS (although this did not reach significance). In combination, these findings suggest that both the activation and the topological organization within the sensorimotor network are affected by rTMS. Future follow‐up studies will be of great value to elucidate the influence of rTMS on the function of the sensorimotor network by combining the resting‐state fMRI and task‐based fMRI.

Our current study has several limitations. First, no significant interaction (frequency of rTMS × MR session) was observed in PSC of each ROI. We conjectured that the parameters of rTMS (stimulation time and the intensity) that adopted in our study may affect its effects. The stimulation time of rTMS might have been too short to detect the alterations in activation within the sensorimotor network. As application of rTMS for the treatment of patients with motor dysfunction always lasts several weeks, future studies with longer stimulation times are required to investigate the effects of rTMS on the activation within the sensorimotor network. Moreover, our study only explored the effects of rTMS with subthreshold intensity, which may be different from the suprathreshold rTMS (Bestmann et al., 2003, 2004; Hanakawa et al., 2009). For future studies, it would be interesting to explore the influence of rTMS with different intensities on the changed activation of the sensorimotor network. Second, previous studies found that the impact of high‐frequency rTMS on brain plasticity was distinct between different frequency rates, such as 3 Hz and 10 Hz (Khedr, Abdel‐Fadeil, Farghali, & Qaid, 2009). Therefore, it will be important for future studies to also examine the effects of these other frequencies on the level of brain activation. Finally, we failed to collect behavioral data in this study. Future studies should explore behavioral alterations in motor function before and after the application of rTMS, which may help us to further understand the relationship between the activation of the sensorimotor network and behavioral alterations.

## 5 CONCLUSION

This study suggested that activation alterations within sensorimotor network are dependent on the frequency of rTMS. Therefore, our findings contribute to understanding the effects of rTMS on brain activation in healthy individuals and ultimately may further help to suggest mechanisms of how rTMS could be employed as a therapeutic tool.

## CONFLICT OF INTEREST

All authors report no conflicts of interest.

## AUTHOR CONTRIBUTIONS

XW, GH, XL, and HM performed data analysis. XW wrote the manuscript. XW, LL, WW, and TZ contributed the acquisition of MRI data and application of rTMS. YL contributed conception of the study and manuscript revision. All authors read and approved the submitted version.

## Supporting information

Supplementary Material

## Data Availability

The data that support the findings of this study are available on request from the corresponding author. The data are not publicly available due to privacy or ethical restrictions.
