# Supplementary material for: Altered activation in sensorimotor network after applying rTMS over the primary motor cortex at different frequencies
Source: Brain Behav. 2020 Jun 7;10(7):e01670. doi: 10.1002/brb3.1670 (PMC7375128; doi:10.1002/brb3.1670)
Supplement: Supplementary file 1 — Supplementary Material [file BRB3-10-e01670-s001.docx]

**Supplementary materials**

**Voxel-Based group differences Analyses**

To validate our findings of the ROI-based analyses, we performed voxel-based analyses on the alterations of activation during LH task and RH task within sensorimotor network.

Voxel-wise one-sample t-tests were performed to generate the group-level activation maps of all participants (n = 45, pre-rTMS) for both LH task and RH task. The sensorimotor mask was then obtained using the unit of two resultant t-maps (*p* < 10^-6^, cluster size > 186 voxels). Voxel-wise between-group differences and within-group differences in activation were inferred within sensorimotor mask. Specially, between-group differences after stimulation were inferred using two-sample t-tests (HF group vs SHAM group, LF group vs SHAM group and HF group vs LF group); within-group (HF group and LF group) differences were inferred with pair t-tests (pre-rTMS condition vs post-rTMS condition). A threshold of *p* < 0.05 (two-tailed) and cluster size > 20 voxels were considered statistically significant.

**RESULTS**

We found that between-group differences and within-group differences remained largely unchanged. Changes in activation were occurred predominantly in several areas: the M1, S1, SMA and premotor cortex.

**Differences within the HF group:** During the LH task, the activation of the left S1 and right PMv was significantly increased after rTMS (*p* < 0.05, cluster size > 20 voxels). Meanwhile, the activation of the left M1 and SMA was increased after the stimulation during the RH task (*p* < 0.05, cluster size > 20 voxels) (Supplementary Figure 1).


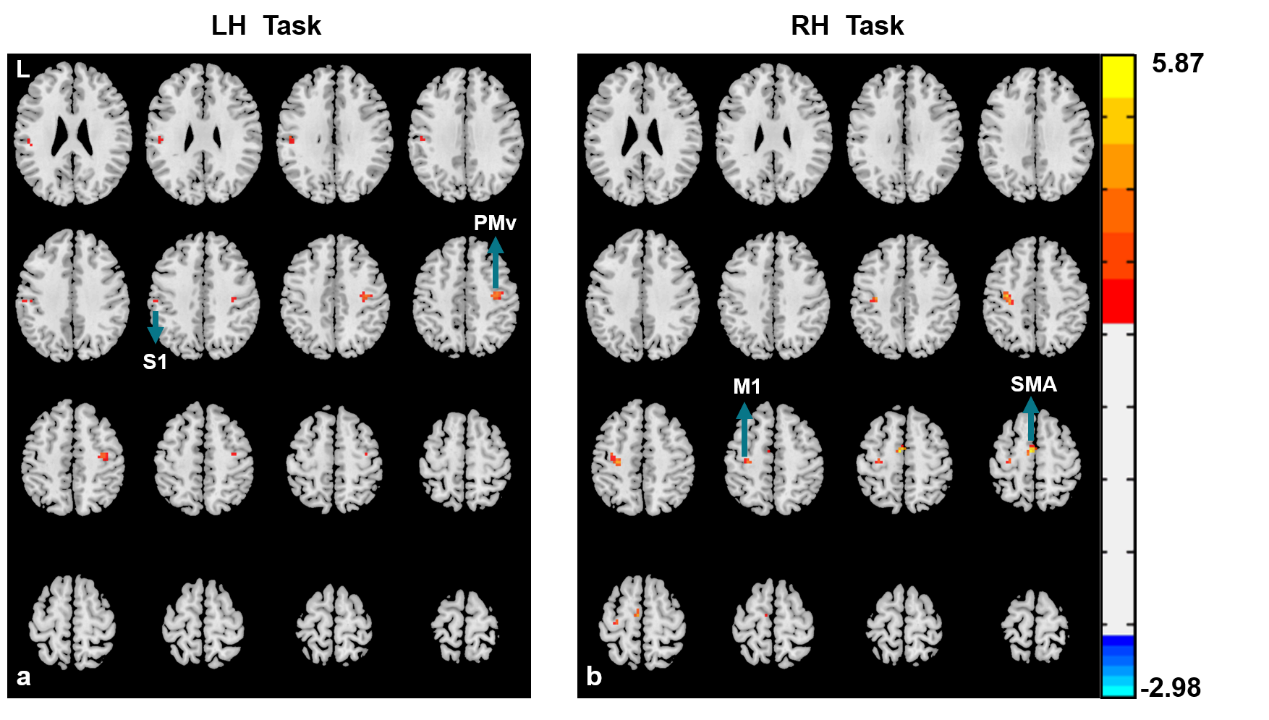


**Supplementary Figure 1** Within-group differences in HF group during left finger-tapping task (a) and right finger-tapping task (b). Warm color denotes the increased activation after high-frequency rTMS. S1, primary sensory cortex; PMv, ventral premotor cortex; M1, primary motor cortex; SMA, supplementary motor cortex.

**Differences within the LF group:** During the LH task, the activation of the left PMv was significantly decreased after the stimulation, while the increased activation was found in the right PMd compared with pre-rTMS condition (*p* < 0.05, cluster size > 20 voxels). The right SMA showed an increased activation during the RH task after the stimulation (*p* < 0.05, cluster size > 20 voxels) (Supplementary Figure 2).


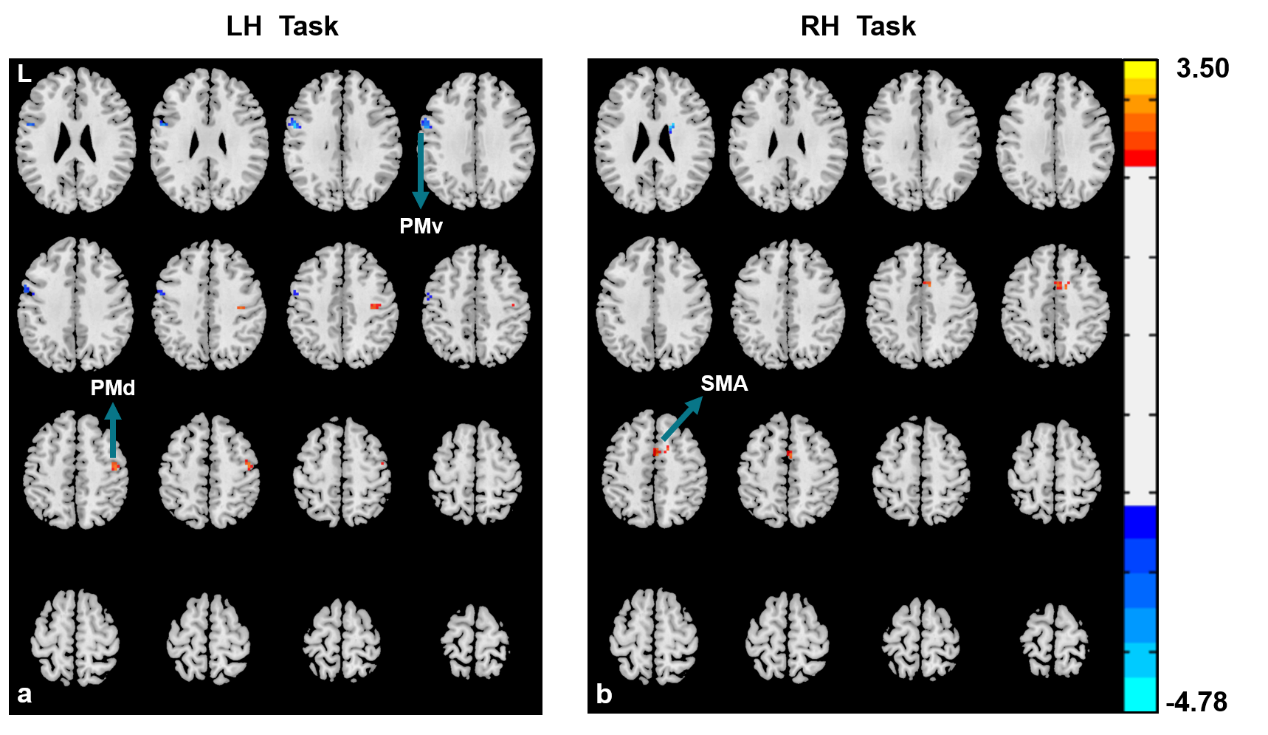


**Supplementary Figure 2** Within-group differences in LF group during left finger-tapping task (a) and right finger-tapping task (b). Warm color denotes the increased activation after low-frequency rTMS. Cold color denotes the decreased activation after low-frequency rTMS. PMv, ventral premotor cortex; PMd, dorsal premotor cortex; SMA, supplementary motor cortex.

**Differences between the HF group and SHAM group:** The activation of the right PMv and SMA was significantly increased during the LH task of the HF group compared with the SHAM group (*p* < 0.05, cluster size > 20 voxels). For the RH task, the activation of the right SMA was also increased significantly in the HF group compared with the SHAM group (*p* < 0.05, cluster size > 20 voxels) (Supplementary Figure 3).


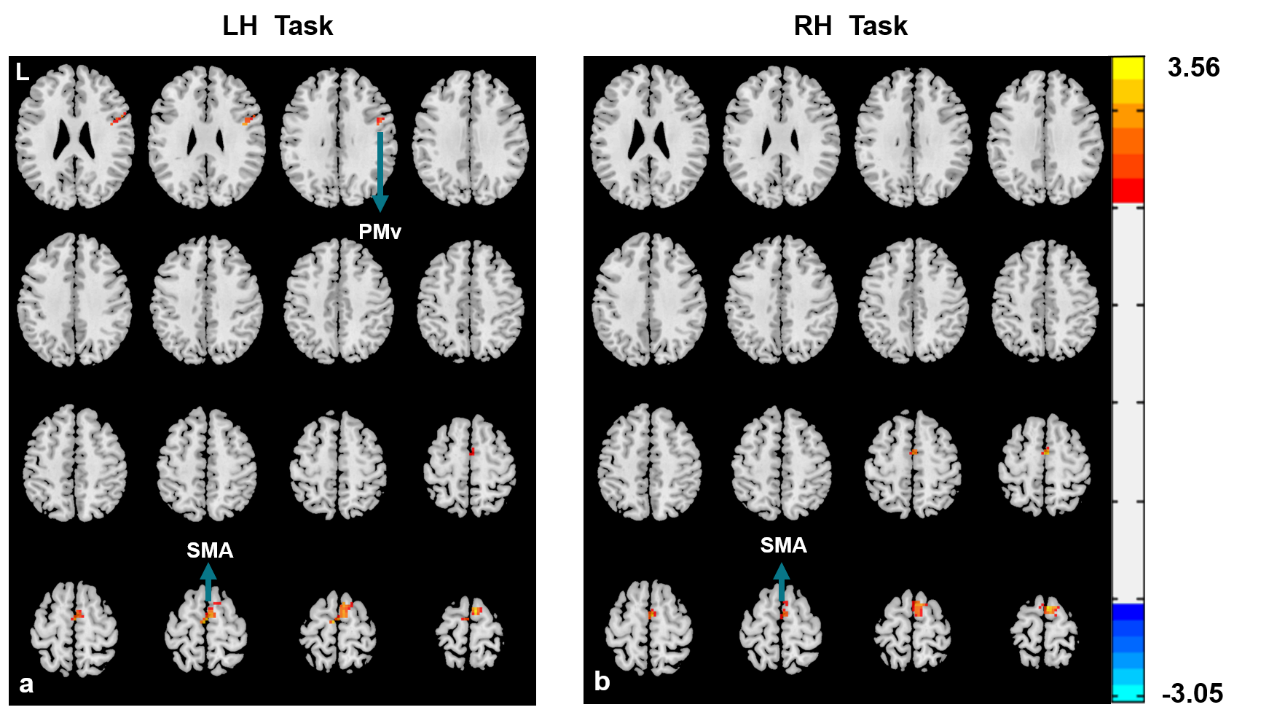


**Supplementary Figure 3** Group differences between HF and SHAM group after stimulation during left finger-tapping task (a) and right finger-tapping task (b). Warm color denotes the increased activation in the HF group compared with the SHAM group. PMv, ventral premotor cortex; SMA, supplementary motor cortex.

**Differences between the LF group and SHAM group:** The deactivation of the right M1 and PMd was significantly increased during the RH task of the LF group compared with the SHAM group (*p* < 0.05, cluster size > 20 voxels). No significant changes were found during the LH task (*p* < 0.05, cluster size > 20 voxels) (Supplementary Figure 4).


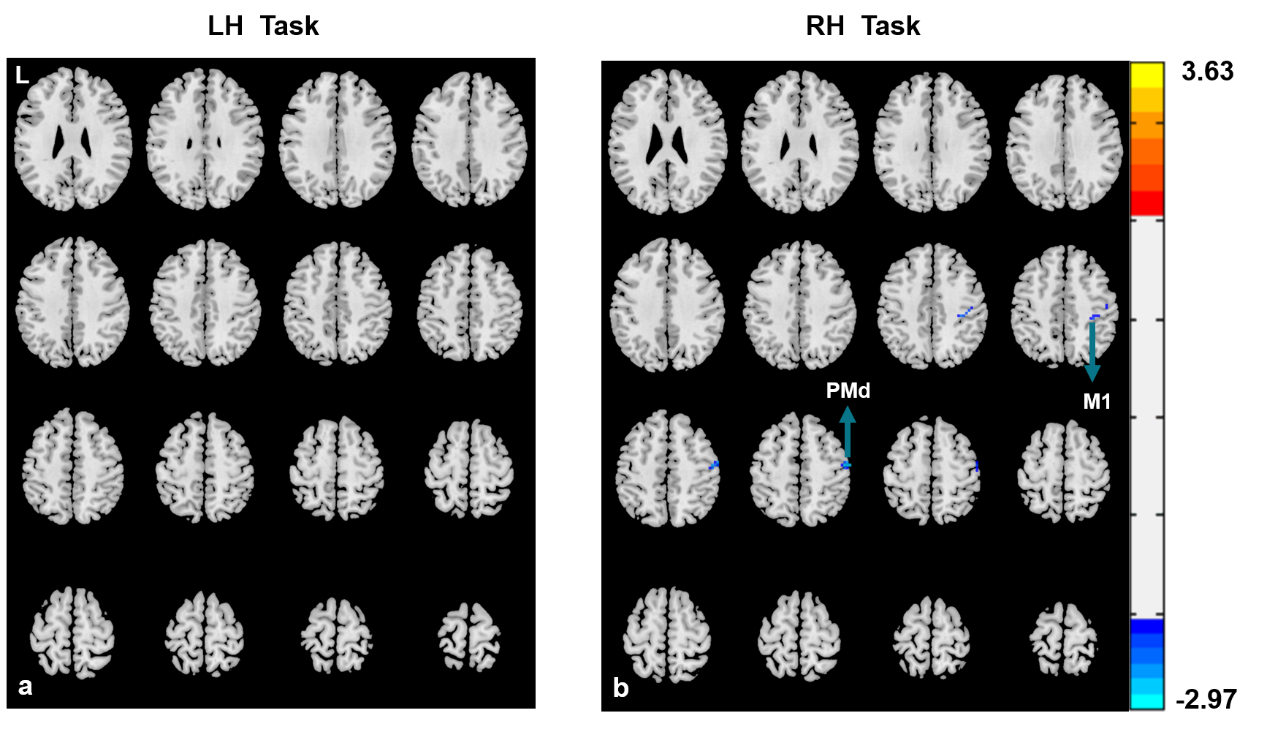


**Supplementary Figure 4** Group differences between LF group and SHAM group after stimulation during left finger-tapping task (a) and right finger-tapping task (b). Cold color denotes the increased deactivation in the LF group compared with the SHAM group. M1, primary motor cortex; PMd, dorsal premotor cortex.

**Differences between the HF group and LF group:** The HF group exhibited an increased activation in the left SMA during the LH task, and the bilateral SMA during the RH task (*p* < 0.05, cluster size > 20 voxels). However, the LF group exhibited an increased deactivation of the right PMd during the RH task (*p* < 0.05, cluster size > 20 voxels) (Supplementary Figure 5).


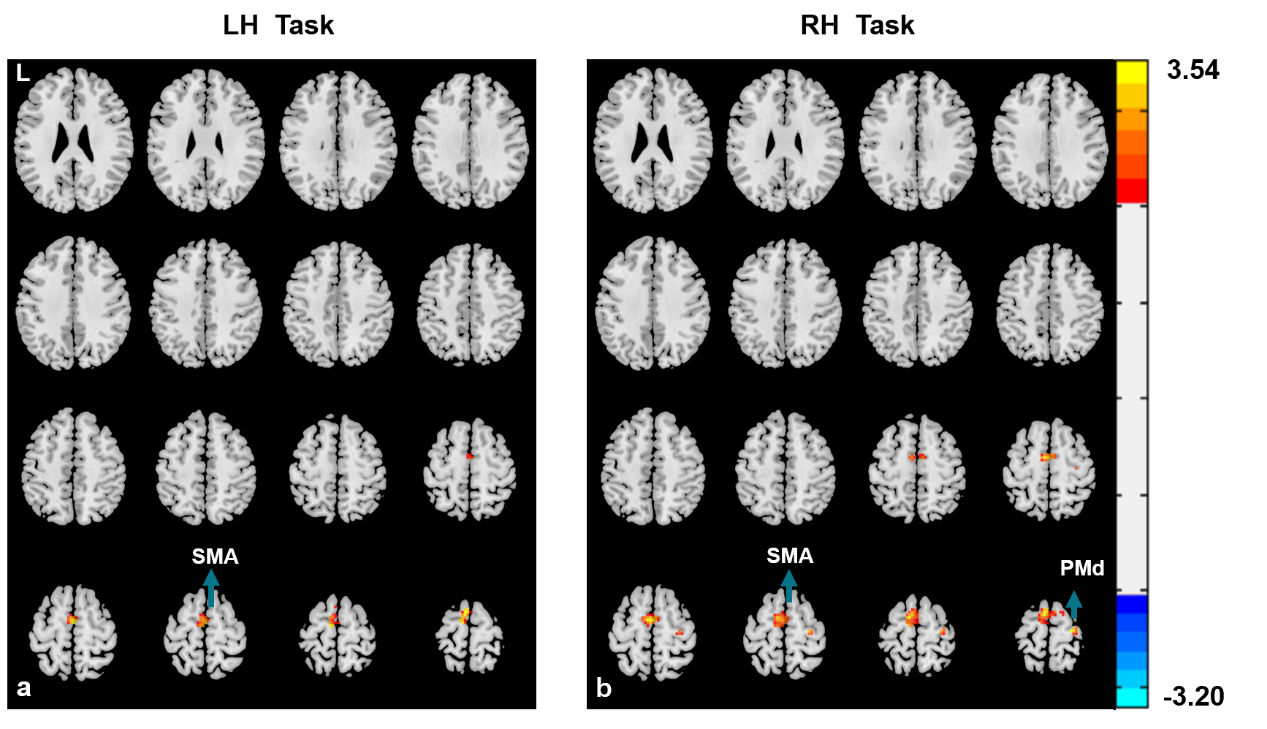


**Supplementary Figure 5** Group differences between HF group and LF group after stimulation during left finger-tapping task (a) and right finger-tapping task (b). Warm color denotes the increased activation or decreased deactivation in the HF group compared with the LF group. SMA, supplementary motor cortex; PMd, dorsal premotor cortex.
